# Draft Genome Sequence of Vibrio parahaemolyticus Strain VP14, Isolated from a Penaeus vannamei Culture Farm

**DOI:** 10.1128/genomeA.00149-18

**Published:** 2018-03-15

**Authors:** Ashok Kumar Jangam, T. Bhuvaneswari, A. Navaneeth Krishnan, Vinaya Kumar Katneni, Satheesha Avunje, Monendra Grover, Sujeet Kumar, S. V. Alavandi, K. K. Vijayan

**Affiliations:** a Nutrition Genetics and Biotechnology Division, ICAR-Central Institute of Brackishwater Aquaculture, Chennai, Tamil Nadu, India; b Aquatic Animal Health and Environment Division, ICAR-Central Institute of Brackishwater Aquaculture, Chennai, Tamil Nadu, India; c Division of Computer Applications, ICAR-Indian Agricultural Statistics Research Institute, New Delhi, India

## Abstract

Here, we report the draft genome sequence of an isolate of Vibrio parahaemolyticus, VP14, recovered from the gut of Penaeus vannamei shrimp farmed in southern India. The genome of VP14 comprised 5,224,046 bp with a GC content of 45.3% and contained 5,326 genes, including 4,972 coding sequences.

## GENOME ANNOUNCEMENT

Vibrio parahaemolyticus is a Gram-negative halophilic bacterium, abundant in brackish water and marine environments. While most strains are nonpathogenic, some cause disease in humans and aquatic animals. Acute hepatopancreatic necrosis disease (AHPND) has emerged as an important disease of concern in the shrimp farming industry, causing substantial economic losses globally (1). AHPND is caused by specific strains of V. parahaemolyticus harboring a plasmid that encodes PirA^vp^ and PirB^vp^ (2). During disease surveillance of farmed shrimp, V. parahaemolyticus was isolated in the Nellore District of Andhra Pradesh, India, from the gut of P. vannamei with reported mortality within 40 days of culture. The purified bacterial isolate was phenotypically identified as V. parahaemolyticus and designated VP14. Further, the species was confirmed by species-specific PCR tests based on the *vpm*, *toxR*, and *gyrB* genes (3) and 16S rRNA gene sequencing. However, this isolate did not reproduce AHPND following previously described experimental protocols (1), and it also failed to produce AHPND-specific PCR amplicons (4). The VP14 genome was found to possess a type III secretin system 1 (T3SS1) based on a positive PCR test for the *vscP* gene. However, a negative PCR test was obtained for the *vscC2, vopB2, vscS2*, and *vopC* (5) genes, which are indicative of the presence of T3SS2. Further, the VP14 isolate could not induce mortality to juvenile shrimp during infection experiments.

To generate the VP14 genome, DNA was initially isolated using a PowerLyzer UltraClean microbial DNA isolation kit (Mo Bio, USA) and sequenced on the Illumina HiSeq platform. The *de novo* assembly with 1,754,630 paired-end reads of 250 bp in the MaSuRCA assembler (6) generated a genome of 5,224,046 bp with a 45.3% GC content. The assembly contained 94 scaffolds with a coverage of 84×, an *N*_50_ length of 279,839 bp, and a mean length of 55,575 bp. Genome annotation was performed with the NCBI Prokaryotic Genome Annotation Pipeline (PGAP) (7) and subsequently with the Rapid Annotations using Subsystems Technology (RAST) server (8). The genome contained 5,326 genes, including 4,972 coding sequences, 10 5S rRNAs, 15 16S rRNAs, 12 23S rRNAs, 161 tRNAs, 4 noncoding RNAs, and 152 pseudogenes.

The VP14 genome was found to have all 39 virulence factors listed as T3SS1 (responsible for causing cytotoxicity of host cells) in the Virulence Factors Database (VFDB, http://www.mgc.ac.cn/VFs), as well as the MAM7 gene (responsible for mediating attachment of bacteria to host cells). However, other virulence factors listed in the VFDB for V. parahaemolyticus were absent. In addition, plasmid-borne genes reported to be associated with AHPND, namely, PirA^vp^ and PirB^vp^, were also found to be absent in VP14. Estimates of average nucleotide identity (ANI) computed for the VP14 genome with other completed genomes of V. parahaemolyticus at GenBank using PyANI (9) indicated its closeness to CHN25, a strain isolated from aquatic products in China, with an ANI value of 98.62%. Analysis of the VP14 genome using ResFinder (10) indicated the absence of any antibiotic resistance genes. The genome of this non-AHPND-causing strain isolated from a brackish water aquaculture system would be an addition to existing genomic resources of V. parahaemolyticus for comparative genomic studies.

### Accession number(s).

The draft genome sequence reported here has been deposited at GenBank under the accession number PKMB00000000.
